# Time and gender measurement invariance in the modified Calderon depression scale

**DOI:** 10.1186/s12955-022-02007-8

**Published:** 2022-06-25

**Authors:** Erika Arenas, Graciela Teruel, Pablo Gaitán-Rossi

**Affiliations:** 1 Department of Sociology, University of California Santa Barbara, Santa Barbara, CA USA; 2 Instituto de Investigaciones Para El Desarrollo Con Equidad (EQUIDE), Universidad Iberoamericana, Mexico City, Mexico

**Keywords:** Depression, Measurement invariance, Confirmatory factor analysis, Mexico, Gender

## Abstract

**Background:**

Assessing change and comparing groups requires high quality and invariant scales. However, there is limited evidence of simultaneous longitudinal and gender measurement invariance for depression scales**.** This evidence is even more scant with long-established panel studies from low and middle-income countries.

**Methods:**

In this paper, we used three waves (years 2002, 2005, and 2009) of a nationally representative panel study to examine the psychometric properties of the modified Calderon Depression Scale (CAL-DM)—a one-item exclusion of a depression scale designed for a population residing in a middle-income country (i.e., Mexico). Our analytical sample included 16,868 participants: 7,696 men and 9,172 women. Using Confirmatory Factor Analysis (CFA), we first examined overall fit in each wave, and then we tested time, gender, and time-gender measurement invariance across three waves. We also estimated and compared depression score means by gender and time. Finally, we examined the association between depression scores and self-rated health.

**Results:**

Our analyses indicated the CAL-DM is a robust scale, suitable for time, gender, and time by gender comparisons. Mean comparisons exemplified how the scale can be used as a latent variable or a summative score. Women have higher depression scores than men and the gap is narrowing from 3.4 in 2002 to 2.5 in 2009.

**Conclusions:**

The CAL-DM is a reliable instrument to measure depression in the Mexican general population that can be used for epidemiological research. Our results will contribute to a burgeoning line of research that examines the social determinants of depression, and the risk factors associated with different individuals’ depression trajectories over the life course.

**Supplementary Information:**

The online version contains supplementary material available at 10.1186/s12955-022-02007-8.

## Background

As of 2017, four percent of the global population suffered from depression (~ 322 millions) [1]. Lifetime prevalence of major depressive episodes ranged from 11.1% in low- and middle-income countries (LMICs) to 14.6% in high income countries [2]. Depressive disorders were among the five leading causes of disability [3] and were associated with higher risks of cardiovascular disease, chronic conditions [4, 5], and all-cause mortality [6, 7]. Over the life course, depressive disorders are common and often recurrent [8]. As world population ages, this trend is expected to rise given that improvements in life expectancy and reductions in mortality will allow more individuals to reach ages when the onset of depression is most common.

During the last two decades, global research initiatives like the World Mental Health Surveys [9], and the Global Burden of Disease Study [10] have increased our understanding of the scope of mental health problems in terms of prevalence, risk factors, and barriers to health care utilization and treatment [2–4, 6, 8, 11–18]. Yet, our knowledge about depression over time continues to be limited and most empirical evidence comes from high income countries [5, 8, 17, 19–21]. Nonetheless, it is on LMICs where more than 85% of the population reside and where individuals are more likely to face poverty, violence, economic inequality, and environmental degradation, all of which are risk factors for depression.

At the population level, research examining trends in depression over time are based either on repeated cross-sectional studies or on panel studies that collect information about depression. Research in this area is hampered for two main reasons. First, cross-sectional studies often do not follow similar sampling procedures and/or assess depression in the same way over time [19]. Second, there are few large scale longitudinal general population studies that collect information about depression using high-quality scales [8]. Data is even more scarce in LMICs, where nationally representative panel studies are largely nonexistent [5, 8, 19].

In this paper we used a long-established nationally representative panel study of the Mexican population to examine the psychometric properties and validity evidence of a depression scale: the modified Calderon Depression Scale (CAL-DM) [22]. We first examined the internal structure of the CAL-DM in three waves to assess unidimensionality and its overall quality. Then, we tested time, gender, and time-gender measurement invariance across three waves of the panel study to show if observed differences are due to measurement bias or if they reflect actual differences in depression [23]. Lastly, we provided convergent validity evidence by comparing the association between the CAL-DM scale and poor self-rated health.

### Calderon’s depression scale (CAL-D)

The CAL-D is a depression scale adapted from the Zung Self-Rating Depression scale (ZSDS) [24]. Replication studies confirmed the validity, reliability and prediction of the ZSDS [25]. Further comparisons using the Minnesota Multiphasic Personality Inventory–2 confirmed the ZSDS had discriminant properties by sex [26]. Doctor Guillermo Calderón Narvaez, born in 1921, and a currently retired prestigious Mexican psychiatrist, modified the ZSDS to ease its clinical use. He argued the scale needed a “no” response option because nonresponse was common and wording was confusing for persons with low educational level [22]. While keeping the same items, these modifications led to the Calderon Depression Scale (CAL-D), which was used primarily in clinical settings, such as the Mexican National Institute of Psychiatry. The advantage of the CAL-D was the inclusion of culture-specific idioms of distress that were not captured by instruments designed for other populations [5, 22, 27, 28]. Similar to the ZSDS and other depression scales, the CAL-D can be applied by non-psychiatrists in epidemiological settings [22].

The CAL-D covers eight of the nine primary depression disorders established in the Diagnostic and Statistical Manual of Mental Disorders (DSM-IV), published by the American Psychiatric Association: depressed mood, diminished interest in sexual activities, poor appetite, insomnia, fatigue, feelings of worthlessness, diminished ability to concentrate, and suicidal ideation (see Additional file 1, part 1, for the questions). Calderón provided evidence of the content validity of the scale and its internal consistency (i.e., Cronbach alpha of 0.86) [22]. The CAL-D was tested in an epidemiological study in Mexico City [29] and several studies found the CAL-D was associated in the expected direction with diverse outcomes, such as suicide attempts [30], diabetes and hypertension [31], family separation [32], social mobility [33], and individual-level multidimensional poverty [34].

The continued use of the CAL-D in clinical settings in Mexico [22] led to its inclusion in the Mexican Family Life Survey (MxFLS), used in this study. However, preliminary analyses of the CAL-D in the MxFLS revealed that item 8—which asked respondents whether they had experienced a decrease in their sexual interest—was not performing as well as in clinical settings nor with the same quality as the other items in the scale. Therefore, to avoid losing more than 25% of the sample and to improve its psychometric performance, we changed Calderon´s scale (CAL-D) by excluding item 8 and renamed the modified version as CAL-DM (an explanation of this decision is in the Additional file 1, part 2). Despite studies showing that CAL-D measures depression, the epidemiological use of the CAL-DM requires further examination of the items´ quality, the different types of invariance, and additional validity evidence, which is the objective of this study.

### Importance of measurement invariance

During the last decade, a burgeoning line of research has focused on understanding the long-term effects of depression over the life course [20, 35–38]. These studies rely on panel data that assess depression across waves, and researchers commonly assume that the same screening instrument captures the construct of interest, “depression”, over time and independently of age. Yet, true changes in symptoms may be confounded with changes in an individual's circumstances [39]. Rather than assuming measures are comparable, it is necessary to empirically test whether the same construct is measured over time i.e., test for time measurement invariance [40]. Unfortunately, population-based studies examining depression prevalence across time rarely test longitudinal measurement invariance [19, 20]. In a meta-analysis aiming at depression change over time in the general population, only 2 out of 17 tested time measurement invariance [41, 42]. Likewise, among twenty studies included in a systematic review on trajectories of child and adolescent depressive symptoms by Shore et al. [20], none of them tested for time measurement invariance. Violation of time measurement invariance can lead to misleading interpretations of depression trends and/or individual’s depression trajectories because observed changes may not necessarily reflect changes in depression, but modifications in respondent’s perceptions about the presence and/or severity of symptoms. These perceptions may be altered by the socioeconomic context where individuals live by altering the frame of reference individuals use to respond to an instrument over time [79].

In terms of gender, previous evidence showed that, in most countries, women were between 2 and 3 times more likely than men to experience major depression [14, 15, 18, 43–49]. The authenticity of gender differences in depression presupposes that this concept was measured in an equivalent way between men and women [50]. However, previous research showed evidence suggesting women were more likely than men to report more symptoms (despite equal social and occupational impairment) [51–53]; and to report higher levels of depressive symptoms [54], thus reaching “caseness” criteria at lower thresholds. Furthermore, women were more likely than men to report certain symptoms, such as tearfulness, experiencing appetite gain and weight changes, disturbances of sleep, fatigue, anxiety, tension, and somatic pain, as well as, feeling self-critical and more irritable [53, 55–59]. If men experience or express depression in a different way that is not asked in such instruments, depression prevalence among men may be systematically underestimated [60, 61]. Research from the United States and Europe suggests gender differences in depression were genuine, even though artefactual determinants may enhance women preponderance in depression [61]. However, few studies in Latin America examined whether this gender gap is present or may be explained by measurement artefacts, and it remains unclear if such a gap persists over time [28, 62, 63].

The aim of the paper was to examine the time, gender, and time-gender measurement invariance of the CAL-DM across three waves of the MxFLS panel study. We expected that the CAL-DM was unidimensional and had adequate psychometric properties in each of the three waves of the MxFLS. Moreover, we hypothesized that—when each pair of waves was compared and when the three waves were tested simultaneously—the CAL-DM was invariant by: (i) time, (ii) gender, and (iii) time-gender. Lastly, we hypothesized higher levels of depression in women, with a stable gap over the study period, and a positive association between poor self-rated health and depression when using latent or total summative scores from the CAL-DM. By demonstrating longitudinal and gender measurement invariance it will be possible to claim that the CAL-DM is consistently measuring the same underlying construct, “depression”, for both men and women, and across time. Given that population-based longitudinal studies in LMICs are scarce, offering validity evidence of the CAL-DM will open unique opportunities to study how socio-economic status, health behaviors, and family and community characteristics are associated to changes in depressive symptoms over time.

## Methods

### Data

Research in Latin America on major depression among the general population relies mostly on cross-sectional data that is not nationally representative [12, 14, 18, 49, 58, 64, 65]. Besides the Chilean Longitudinal Survey [66], the Mexican Family Life Survey (MxFLS) is the only panel study representative of the general population [67] covering the life span, thus allowing the investigation of the cumulative effects of depression over the life course. Until now, none of these sources has tested time nor gender measurement invariance in their depression scales.

We examined the psychometric properties of the CAL-DM using data from the MxFLS. The baseline survey consisted of a stratified random sample of the Mexican population representative at the national level. MxFLS-1 was conducted in 2002 and included approximately 8,400 households and 35,000 respondents. MxFLS-2 and MxFLS-3 were conducted in 2005–2006 and 2009–2012, respectively; for brevity, waves were labelled as “2002”, “2005”, and “2009”. Re-contact rates for MxFLS-2 and MxFLS-3 were about 90% [67, 68].

The present analysis used three waves of the MxFLS. The age-eligible sample included original members of the survey, who were 15 years or older at baseline and who were alive when MxFLS-3 was conducted; it excluded migrants to the United States. Our sample consisted of 21,635 individuals. Of these individuals, we dropped cases who did not answer the mental health module because they refused to be interviewed (n = 1052), their households were not found (n = 1370), or they were not present at the time of the interview (n = 554) further reducing the sample to 18,659. Finally, we dropped cases with missing values in any of the variables used in the analysis (n = 1791) leading to an analytical sample of 16,868 respondents; 7696 men and 9172 women. An analysis testing if those in the analytical sample differed from those not included revealed that, as individuals age, they were more likely to be in the sample and individuals with higher education and residing in women-headed households were less likely to remain in the sample.

### Measures

The MxFLS mental health module asked the original 20 questions included in the instrument designed by Calderón [22]. These items –labelled in the analysis as d1, d2,…d20—inquire about the presence and severity of behaviors and feelings four weeks prior to the interview, with possible answers being 1. *No, 2. Yes sometimes, 3. Yes lots of times, or 4. Yes all the time*. Preliminary analyses evidenced that item 8 had the highest proportion of missing values (15% in 2002, 25% in 2005, and 17% in 2009), the lowest patterns of inter-item polychoric correlations, and the weakest relationship with the latent variable “depression” (i.e., low factor loading). Therefore, we excluded this item from the analysis. Besides these analyses, Additional file 1: Table S1 , part 2, shows measures of fit for the original CAL-D per wave. These results show the CAL-D, in its original version, has a good-enough fit. However, instead of using a 20-item scale, we estimated a summative score by adding responses of the remainder 19 questions to preserve most of the sample and a better fit of the scale. Thus, the modified Calderón Scale, the CAL-DM, is the same scale but without item 8. Poor self-perception of health (SRH) was generated as an indicator equal to one when respondents answered their health status was “*Regular*”, “*Bad*” or “*Very Bad*” and zero when they answered it was “*Very good*” or “*Good*”.

### Analytical strategy

The objective of measurement invariance was to assure that the probability of selecting a particular response option, for any individual item, was the same across time and between groups, given the same standing on a common factor (i.e., depression) [69]; therefore, men and women with the same level of depression should score equally on every item on the three waves. Absence of measurement invariance implies latent variable comparisons by time and/or gender are not valid.

We tested measurement invariance using Confirmatory Factor Analysis (CFA), which allowed establishing invariance in multiple parameters [70]. CFA measurement models for ordinal data estimated several parameters: (1) factor loadings which can be interpreted as weighted slopes (i.e., higher values indicated the latent variable had a stronger association with the item); (2) item intercepts, representing the mean levels of each item; (3) three item-response thresholds (each threshold labelled t1, t2, t3) indicating where the observed responses cross-over from one response category to the next; (4) item residuals, which represented unexplained variance from the latent variable and were assumed to be independent (lower values indicated lower measurement error) [71]. In addition, mean differences and predicted scores between groups were computed (i.e., between waves and gender).

Measurement invariance assessment was conducted by comparing the fit of a series of nested models, each of them with harsher constraints on the model parameters [72]. The baseline model, named the configural model, tested whether the unidimensional factor structure of the scale was equal between time-waves and/or men and women. The configural model was the least stringent because it only demanded unidimensionality—for all items to load into one latent variable, allowing factor loadings, item intercepts, thresholds, and residuals to vary freely. Sequential constraints on the model parameters were then added to compare multiple degrees of invariance. We used the Wu and Estabrook [74] identification strategy by fixing, first, item thresholds –treating it as the baseline model, then fixing item loadings, followed by item intercepts, and finally the residuals [73]. This modification allowed us to test all parameters at the same time, achieving invariance when thresholds, loadings, and intercepts were equal (i.e., when setting the same values on each parameter did not significantly decrease model fit). Only when full invariance was achieved (i.e., including item residuals) summative means of observed items were used to compare depression across time and gender [74].

The analysis was conducted in sequential steps to examine multiple combinations of measurement invariance and thus facilitated the identification of focal points of invariance. We first examined single, separate, CFAs for each of the three waves to examine overall quality and unidimensionality of the configural model. Then we assessed time measurement invariance in each pair of the three MxFLS waves, and then the three waves simultaneously. Next, we examined time and gender invariance altogether; again, we first compared each pair of waves and then the three waves together while accounting for gender. In this paper, we only present model parameters of the fully invariant model by time and gender; other analyses are in the Additional file 1.

We began fitting ordered CFA models with mean and threshold structures using the Diagonally Weighted Least Squares (DWLS) method and theta parameterization to consider the unique variances as model parameters. Latent means and variances were fixed to 0 and 1 to scale the baseline model. All models used sampling weights and pairwise deletions, so sample size varied slightly in each model. We included the Satorra-Bentler scaling correcting factor and robust measures of fit. For group comparisons, the reference category was the men’s latent mean of depression; for the time-wave comparisons the reference category was the latent mean during the 2002 wave. All results were computed using *R software* [75] with the *cfa* command in the *lavaan* package [76]. We relied on the *measEq.syntax* from *semTools* [77] to follow the "Wu.Estabrook.2016'' strategy to identify model parameters [78].

We assessed model fit with absolute and comparative indices [70] using common cutoff criteria: non-significant Chi-Square; RMSEA values below 0.06; SRMR values below 0.08; and CFI and TLI values of 0.95 or greater [78]. For nested models, we used: 0.015 in ΔRMSEA; 0.01 ΔCFI and ΔTLI; and 0.03 in ΔSRMR [79] (results available upon request).

We complemented the analysis with mean comparisons using the fully invariant model by time and gender. Specifically, we used predicted latent scores and summative scores to compare means between time and gender. Finally, we compared summative scores with poor SRH to provide convergent validity evidence of the CAL-DM.

## Results

Women, compared to men, consistently reported higher values for the summative score of the 19-item CAL-DM scale (26.1 vs. 23.3 points); a consistent 2.3 to 3.3 percentage point difference across waves. Compared to men, the percentage of women reporting poor SRH (52.3% vs. 44.4%) was consistently higher across waves, with a difference between 7.5% and 8.3% (Table 1).

**Table 1:** Descriptive statistics by time and gender (n = 16,868)

Wave	Gender (%)		CAL-DM summative (mean, SD)		Poor SRH (%)
2002	Women	58.27	26.9	7.29	51.84
	Men	41.73	23.5	5.36	43.58
2005	Women	58.47	25.5	7.5	52.17
	Men	41.53	23.1	6.33	44.44
2009	Women	58.91	25.8	7.84	52.78
	Men	41.09	23.3	6.28	45.19
Total	Women	58.54	26.1	7.57	52.25
	Men	41.46	23.3	6.01	44.38
	Total	100	24.9	7.03	48.99

*n*  sample size; *CAL-DM* Calderon depression scale modified, summative score; SD standard deviation; *SRH*  Self-reported health status. The minimum value for the summative score is 19 and the maximum is 76.

### Single-wave confirmatory factory analysis

The CAL-DM had adequate psychometric properties when each wave was assessed separately. Results from single CFAs of the CAL-DM showed that all factor loadings and thresholds were statistically significant and with similar values. Moreover, the scale was unidimensional, with all models indicating an adequate fit; RMSEA was below 0.043 in every wave, CFI and TLI above 0.99, and the highest SRMR was 0.049 (see Additional File 1: Tables S2–S4).

### Time invariance analysis

The second set of results demonstrated the CAL-DM was time invariant. Models had adequate fit when separate pairs were compared—2002 vs. 2005; 2002 vs. 2009; 2005 vs. 2009 (see Additional file 1: Table S5). Importantly, longitudinal invariance held when measured simultaneously in the three waves (see Additional file 1: Table S6). Therefore, time comparisons with the 19-item CAL-DM scale were warranted—even with summative scores. Latent mean differences decreased by 0.357 of a standard deviation between 2002 and 2005. However, this difference was smaller, 0.234 of a standard deviation, between 2002 and 2009.

### Time and gender measurement invariance analysis

The next set of results tested time and gender measurement invariance simultaneously. Consistent with previous results, the 19-item CAL-DM scale had adequate fit (Table 2). Chi-Square remained significant in all models. As expected, RMSEA was low, ranging from 0.018 to 0.020, and SRMR from 0.041 to 0.043. Comparative indices had high values; CFI and TLI ranged between 0.990 and 0.992. Differences in model fit were negligible and there were no concerning points of local misfit. Results showing adequate fit for each pair of waves are available in the Additional file 1: Table S7. The model with constrained thresholds, loadings, intercepts, and residuals was the most stringent (labeled as “Residuals” in Table 2). As with previous models, despite a significant chi-square and chi-square difference, it had an adequate fit: RMSEA = 0.020, CFI/ TLI = 0.99, and SRMR = 0.043. Thus, comparisons by time and gender are justified.

**Table 2:** Model fit indices of the time and gender invariance models comparing the three waves of the MxFLS (Men = 7696; Women = 9172)

Invariance Model	SB-Chisq	df	RMSEA	CFI	TLI	SRMR	dchi	ddf	P-value
Configural	11,690.73	2958	0.019	0.992	0.991	0.041	–	–	–
Thresholds	11,800.64	3053	0.018	0.992	0.991	0.041	241.893	95	0
Loadings	11,982.25	3143	0.018	0.992	0.992	0.041	163.42	90	0
Intercepts	12,865.55	3233	0.019	0.991	0.991	0.041	661.984	90	0
Residuals	14,181.73	3328	0.020	0.990	0.990	0.043	433.459	95	0

We report robust fit indices. *SB-Chisq*  Satorra-Bentler Chi-square; *df* degrees of freedom; *RMSEA* Root mean square error of approximation; *CFI* Comparative fit index; *TLI* Tucker-Lewis Index; *SRMR* Standardized root mean square residual; *dchi* Chi-square difference; *ddf* difference in degrees of freedom; *P-value*  *P*-value of the Chi-square difference.

**Fig. 1:**
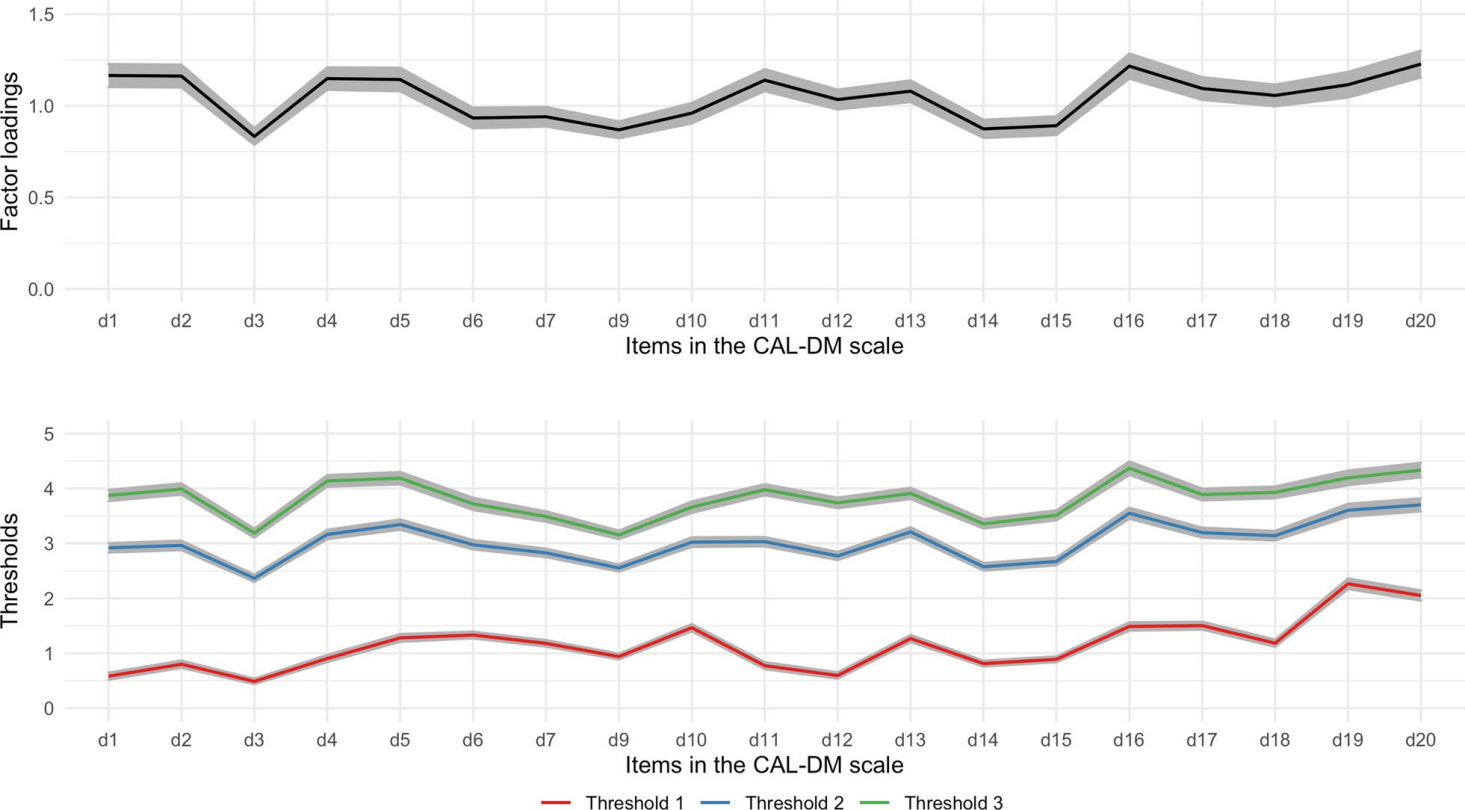
Factor loadings and threshold parameters of the CAL-DM from the fully invariant model by time and gender. Colored lines indicate parameter values and shaded areas show confidence intervals. The unstandardized parameters are the results of the “Residuals” model in Table 2. Note that item 8 is missing because it was excluded from the analyses

Given that we found measurement invariance across waves and gender, Fig. 1 shows fixed values of factor loadings and thresholds corresponding to the “Residuals” model from Table 2—the most stringent, the fully constrained invariant model. Unstandardized factor loadings for each item were similar in strength (range: 0.833–1.228), indicating all items had a strong and equivalent association with the latent variable of depression. Likewise, the threshold parameters resembled each other and did not overlap. The distance between threshold 1 and threshold 2 was larger than between threshold 2 and threshold 3, showing the first gap was slightly better at discriminating the intensity of the latent variable. Items 19 and 20 were the most severe questions to endorse: “Do you wish to die?” and “Do you feel apathetic, without interest in things?”, correspondingly (see unstandardized parameters in Additional file 1: Tables S8 and S9).

**Fig. 2:**
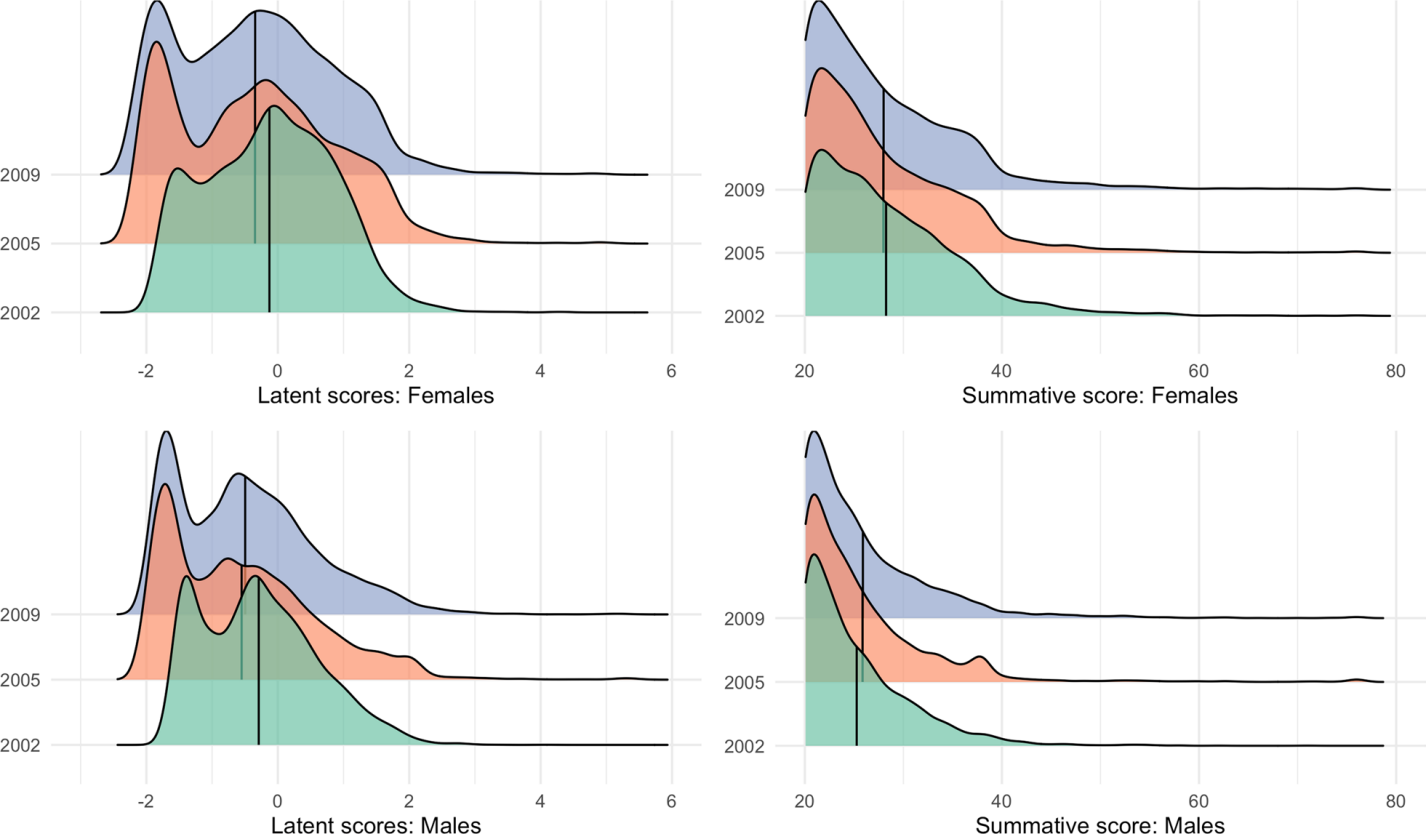
Latent and summative score distributions of the CAL-DM by gender in the three waves of the MxFLS. Upper blue areas correspond to the 2009 wave, middle, orange, areas to the 2005 wave, and lower, green, areas to the 2002 wave. Vertical lines across the distributions show the mean

When estimated as a latent variable or as a summative score, women scored higher than men for almost half a standard deviation. The difference was widest in 2002 and narrowed with time until 2009. Put differently, in a summative score between 19 and 76, the difference between men and women amounted to 3 points—a little lower than half a standard deviation—and the gap was narrowing over time, from 3.4 in 2002 to 2.5 points in 2009 (Table 3). Notably, these gender differences suggest that the women to men ratio is lower than 2:1, as has been reported in other countries (2). In men, 2002 was the wave with the highest latent scores; both differences—of 0.260 between 2002 and 2005 and of 0.206 between 2002 and 2009—were statistically significant, but small (less than a point in the summative score). The latent score had a small and significant increase of −0.054 between 2005 and 2009 (Table 3). In women, 2002 also showed the highest values; differences were moderate (above one summative point) and significant, 0.219 and 0.217, respectively. However, the difference between 2005 and 2009 was not statistically significant. Summative score differences told a similar story, albeit with different distributions (Fig. 2).

**Table 3:** Means and mean differences of predicted latent scores and summative scores by gender and time

	Means of predicted latent scores Mean (standard deviation)				Means of summative scores Mean (standard deviation)			
	All	2002	2005	2009	All	2002	2005	2009
Men	−0.471 (1.120)	−0.290 (0.878)	−0.550 (1.130)	−0.496 (1.060)	23.3 (6.01)	23.5 (5.36)	23.1 (6.33)	23.3 (6.28)
Women	−0.293 (1.170)	−0.128 (0.963)	−0.347 (1.210)	−0.345 (1.180)	26.1 (7.57)	26.9 (7.29)	25.5 (7.50)	25.8 (7.84)

	Difference of latent scores Mean difference [CI]				Difference of summative scores Mean difference [CI]			
			2002 vs 2005	2002 vs 2009			2002 vs 2005	2002 vs 2009
Men			0.260	0.206			−0.451	−0.223
			[0.228; 0.291]	[0.176; 0.237]			[−0.658; −0.248]	[−0.420; −0.022]
Women			0.219	0.217			−1.37	−1.05
			[0.189; 0.251]	[0.185; 0.248]			[−1.59; −1.15]	[−1.27; −0.815]

				2005 vs 2009				2005 vs 2009
Men				−0.054				0.227
				[−0.088; −0.020]				[−0.004; 0.448]
Women				0.002				0.324
				[−0.037; 0.031]				[0.083; 0.564]

Values between parentheses are standard deviations. Values among brackets show confidence intervals. Latent scores are weighted scores by factor loadings and thresholds parameters estimated using the “Residuals”, and summative scores add up equally weighted items.

For the latent scores, minimum value is −2.24 and maximum is 5.47; and for the summative score minimum value is 19 and maximum is 76.

### Evidence of convergent validity with self-rated health (SRH)

**Fig. 3:**
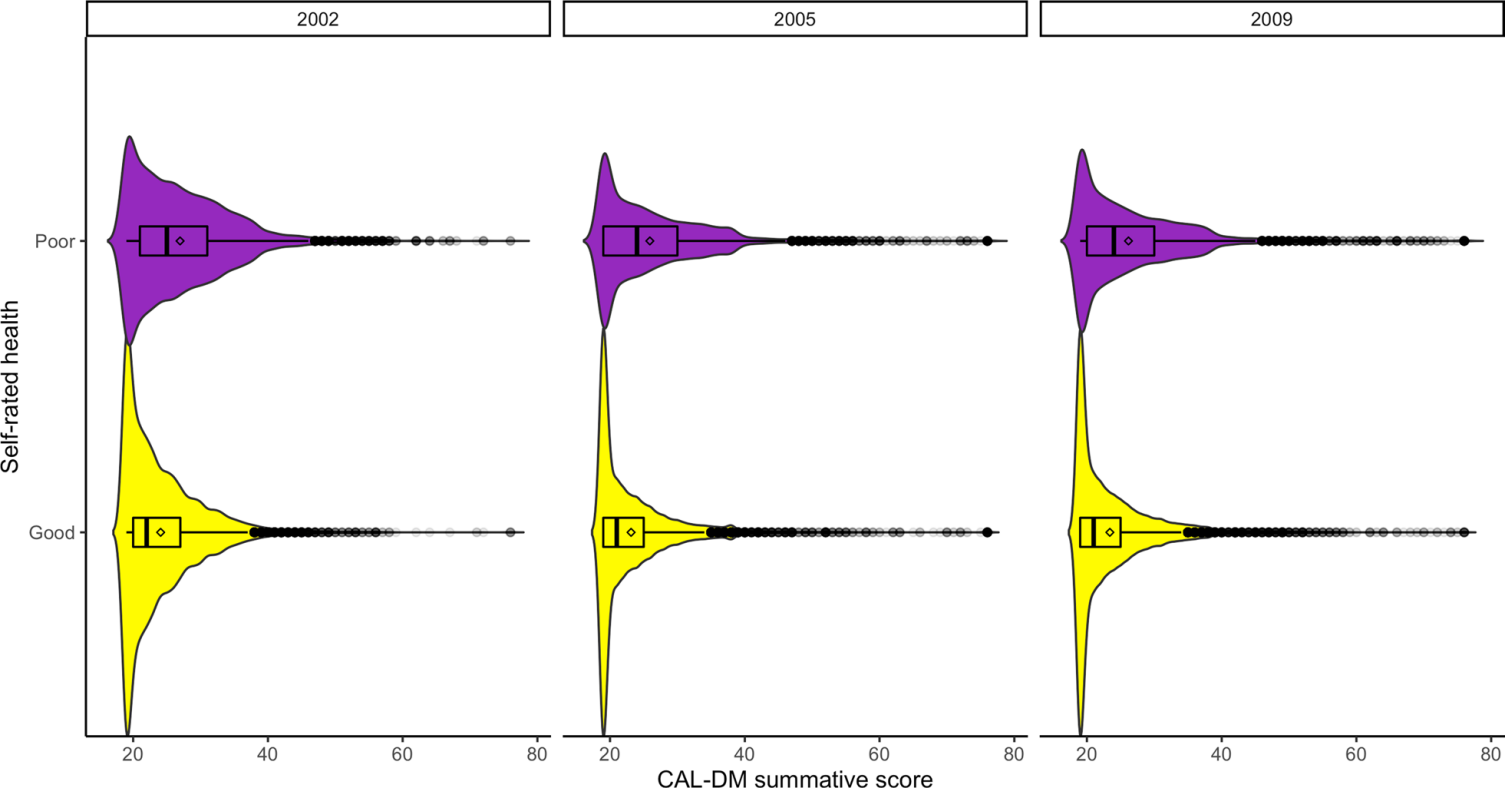
Comparison of summative score distributions of the CAL-DM by self-rated health in the three waves of the MxFLs. Upper purple distributions indicate “Regular”, “Bad” and “Very Bad” self-rated health (labelled “Poor”), while lower yellow distributions reflect “Very good” and “Good” self-rated health (labelled “Good”). Boxplots within the distributions show the median with a vertical line and the mean with a square

To assess convergent validity of the CAL-DM we hypothesized that persons reporting poor SRH would be more likely to report higher values in the CAL-DM summative score. On average, persons with good and very good SRH scored 2.82 points lower in the CAL-DM summative score compared to those with poor SRH [CI 95% 2.75; 2.90]. Figure 3 illustrates this association across the three waves. As expected, we found that most respondents with good and very good SRH showed a depression score of 19—the lowest possible—across the three waves. By contrast, respondents reporting poor SRH were more likely to show higher CAL-DM summative scores. These results add to the validity evidence of the CAL-DM scale.

## Discussion

The design and evaluation of effective public health policies intended to reduce depression prevalence requires information about depression variation and change. A prerequisite for this research is to establish that the underlying construct that we attempt to measure (i.e., depression) is consistently measured over time and across groups (e.g., gender) [80]. Our analyses indicated that the CAL-DM is a reliable instrument to measure depression in the Mexican general population that can be used for epidemiological research. Time, gender, and time-gender measurement invariance models showed adequate model fit with strict constraints; factor-loadings, thresholds, intercepts, and residuals. Our models yielded consistent results along different combinations using samples from three waves of the MxFLS (2002, 2005, and 2009). The CAL-DM proved to be a robust scale and suitable for time, gender, and time-gender comparisons.

The study also added to the validity evidence of the CAL-DM. The CAL-D is based on the well-established ZSDS scale [25, 26]. Modifications in the response options and the wording of the scale led to the CAL-D, which was mostly used in clinical settings in Mexico [22]. Even though the CAL-D was shown to have content validity [22] and it remained closely aligned to the DSM-IV guidelines, the structure of CAL-D was insufficiently tested, especially its factor structure [29]. A new modification of the scale (i.e., dropping item 8), the CAL-DM, required additional evidence of its unidimensional structure, item performance, and its validity. In this study, three separate single CFAs without constraints showed good fit, indicating that the CAL-DM was unidimensional in three samples. Moreover, in all models every item was statistically associated with the latent variable and items had standardized factor loadings above 0.65. The association with SRH amounts to the evidence that the CAL-DM is indeed measuring the construct of “depression”. The CAL-D was adjusted from the ZSDS to facilitate the understanding of the items by low-educated Mexican patients in clinical settings [22]. However, these items could be used in other Spanish-speaking countries, especially in Latin America, because they list symptoms closely aligned to DSM guidelines. Importantly, our results showed that these studies may be conducted within a latent variable framework –as in structural equation models, where measurement error is accounted for—or with a summative score—as is more common in applied research and clinical settings.

A remarkable aspect of the time invariance results is that the measurement quality of the CAL-DM endured for nearly a decade—a feature rarely examined in the Latin American region because of the scarcity of long panel studies including such detailed depression scales. A limitation of the present study was that our analyses relied on data collected 13 years ago. Nonetheless, given the scarcity of panel data from LMICs, the MxFLS constitutes a unique source for longer life-course analyses of depression, more so with every new wave of data collection (i.e. MxFLS-4 will cover a 20 year span). However, a key dilemma for multi-thematic panel surveys along extended periods of time—as the MxFLS—is whether to maintain the original scales to ensure comparability or to replace them with novel scales that adhere to current standards (i.e., the DSM) or to dominant scales (i.e., CES-D). Notably, the time invariance of the CAL-DM means that all the items of the scale remained relevant with cohorts with a similar age-structure but 10 years apart; thus, evidencing that the CAL-DM is useful for long-term comparisons.

Another limitation of the analysis is the lack of a criterion or gold-standard variable in the MxFLS to help define an epidemiological cut-off point for the CAL-DM. Modifications in the DSM and the new discoveries in the field of depression also imply that measures that were validated in the mid-nineties need to be re-examined with current criteria (i.e., items could be losing relevance). A common strategy to update the validity of the CAL-DM is comparing the scale´s scores with clinical interviews and/or well-established depression scales using a sub-sample of the larger study. However, recent and promising developments show that functional neuroimaging, particularly functional near-infrared spectroscopy (fNIRS), can serve as a direct and objective measure of major depressive disorder [81] and can be used as a criterion variable to renew the validation of the CAL-DM. A key advantage of fNIRS is the possibility to disentangle mental health diagnoses with similar symptoms because scales such as the CAL-DM might confound depression with bipolar disorder and borderline personality disorder [82]. Moreover, novel analytic techniques (i.e., machine learning) can use the fNIRS measures to identify the most predictive items and optimal cut-off points for the CAL-DM [83]. Such measurement improvements in future studies will advance the understanding of depression in two important directions. First, they will make scales as the CAL-DM more insightful for clinical practice. Testing the CAL-DM in clinical settings needs to be updated with more relevant measures stemming from the Clinimetric approach, which focuses on the clinical utility of the rating scales and, with novel metrics, provides insights on types of symptoms, staging of illness, remission, and recovery [84]. Second, as the equipment needed to collect fNIRS is becoming smaller [85], easier and cheaper to use during fieldwork, these studies can help build the foundation to change the measurement standard for epidemiological studies. Just as the spectrometer deepened the discussions on how to best measure skin color [86], fNIRS has the potential to reveal limitations of popular depression scales and change standard practice in data collection.

Given that the CAL-DM is embedded in a population-based longitudinal study, establishing time and gender measurement invariance lays the groundwork for the analysis of how greater exposure to violence, poverty, and/or socioeconomic inequality are likely to lead to different depression trajectories. A promising area of inquiry is the identification of social determinants of mental health—like depression—in LMICs. Previous research has shown the importance of socioeconomic variables to understand variations in depression, but studies from Latin America remain scarce. Recently, high quality depression scales revealed key differences between countries in Asia during the COVID-19 pandemic [87]. Available probabilistic datasets that include the CAL-DM can be used as baseline measures to assess change when a traumatic event occurs, such as a disaster. Our results on the quality of the CAL-DM could also encourage investigators to contribute to the growing line of research examining population heterogeneity on individuals’ depression trajectories over the life course [20]. Comprehensive and longitudinal surveys like the publicly available MxFLS are ideal to move these research agendas. The present study is timely because the fourth wave of the MxFLS will soon be published, and a new generation of researchers will benefit from a wealth of data covering 20 years. Our results will contribute to widen research opportunities to study depression over the life course with panel data and a high-quality depression scale.

## Conclusions

Depression is a major public health concern affecting the quality of life of millions worldwide (1). The design and evaluation of effective public health policies requires information about depression trajectories over time and across groups. We provided evidence on the quality of the CAL-DM using a representative sample of Mexico along three waves spanning over a decade. We showed the scale has consistent psychometric properties and is invariant by time, gender, and time-gender. It is thus suitable for long-term comparisons using a latent variable and a summative score. The CAL-DM is a useful scale to study the long-term determinants of depression within a large-scale and multi-thematic panel survey as the MxFLS.

## Supplementary Information

**Additional file 1**. Supplementary Material.

## Data Availability

Data and documentation for the three waves of the MxFLS are publicly available at the project´s website (www.ennvih-mxfls.org). Analytical sample and computer code to reproduce the analyses for this study are available by emailing the corresponding author.
