# Supplementary material for: Time and gender measurement invariance in the modified Calderon depression scale
Source: Health Qual Life Outcomes. 2022 Jun 25;20:100. doi: 10.1186/s12955-022-02007-8 (PMC9233789; doi:10.1186/s12955-022-02007-8)
Supplement: Supplementary file 1 — Additional file 1. Supplementary Material. [file 12955_2022_2007_MOESM1_ESM.docx]

**Supplementary material**

We present additional information for the article “Time and Gender Measurement Invariance in the Modified Calderon Depression Scale (CAL-DM)”.

**1. Items of Calderon Depression Scale – CAL-D (Spanish/ English)**

*“The following questions make reference to how you have felt in the last four weeks. Please specify if you feel that way sometimes, many times, all the time or definitely no.”*

1. ¿Se siente triste o afligido? / Do you feel sad or sorrowful?

2. ¿Llora o tiene ganas de llorar? / Do you cry or do you feel like crying?

3. ¿Duerme mal de noche? / Do you sleep badly at night?

4. ¿En la mañana se siente peor? / Do you feel worse during the mornings?

5. ¿Le cuesta trabajo concentrarse? / Do you find it hard to focus?

6. ¿Le ha disminuido el apetito?* / Have you lost your appetite?

7. ¿Se siente obsesivo o repetitivo? / Do you feel obsessed or repetitive?

8. ¿Ha disminuido su interés sexual? / Has your sexual interest diminished?

9. ¿Considera que su rendimiento en el trabajo o en el estudio es menor? / Do you consider that your performance at your work or studies has decreased?

10. ¿Siente presión en el pecho? / Do you feel a pressure in your chest?

11. ¿Se siente nervioso, angustiado o ansioso? / Do you feel nervous, worried or anxious?

12. ¿Se siente cansado o decaído? / Do you feel tired or downcast?

13. ¿Se siente pesimista, piensa que las cosas le van a salir mal? / Do you feel pessimistic, think that things will go badly?

14. ¿Le duele con frecuencia la cabeza o la nuca? / Does your head or nape hurt often?

15. ¿Está más irritable o enojón que antes? / Are you more irritable or grouchy than before?

16. ¿Se siente inseguro, con falta de confianza en usted mismo? / Do you feel insecure, with lack of trust in yourself?

17. ¿Siente que le es menos útil a su familia? / Do you feel like you are less useful to your family?

18. ¿Siente miedo de algunas cosas? / Are you afraid of certain things?

19. ¿Siente deseos de morir? / Do you feel a desire to die?

20. ¿Se siente apático, sin interés en las cosas? / Do you feel apathetic, without interest in things?

**2. Item 8 removal**

Item 8 was removed from all the analyses in the paper. First, it has considerably more missing values than any other item: 1,615 in the 2002 wave, 2,788 in the 2005 wave, and 2,054 in the 2009 wave. Second, it has the lowest polychoric correlations between the items of the CAL-D (i.e. the lightest blue in the three matrices below), indicating a weaker intra-item association. Third, item 8 has the lowest factor loadings in the single CFAs; it is consistently below .7 in the three waves (see graph at the end). Thus we suggest estimating the CAL-D without item 8.


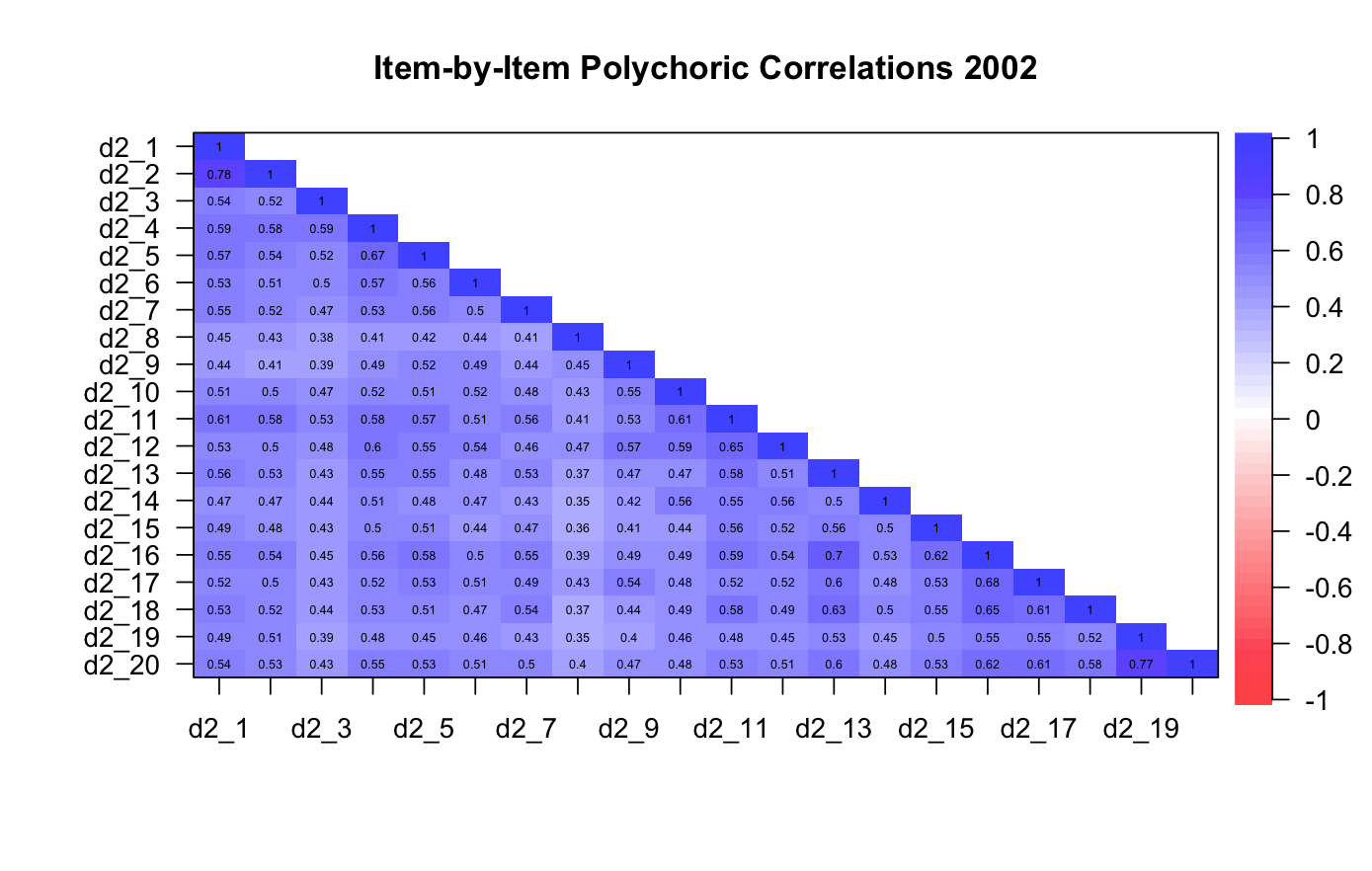


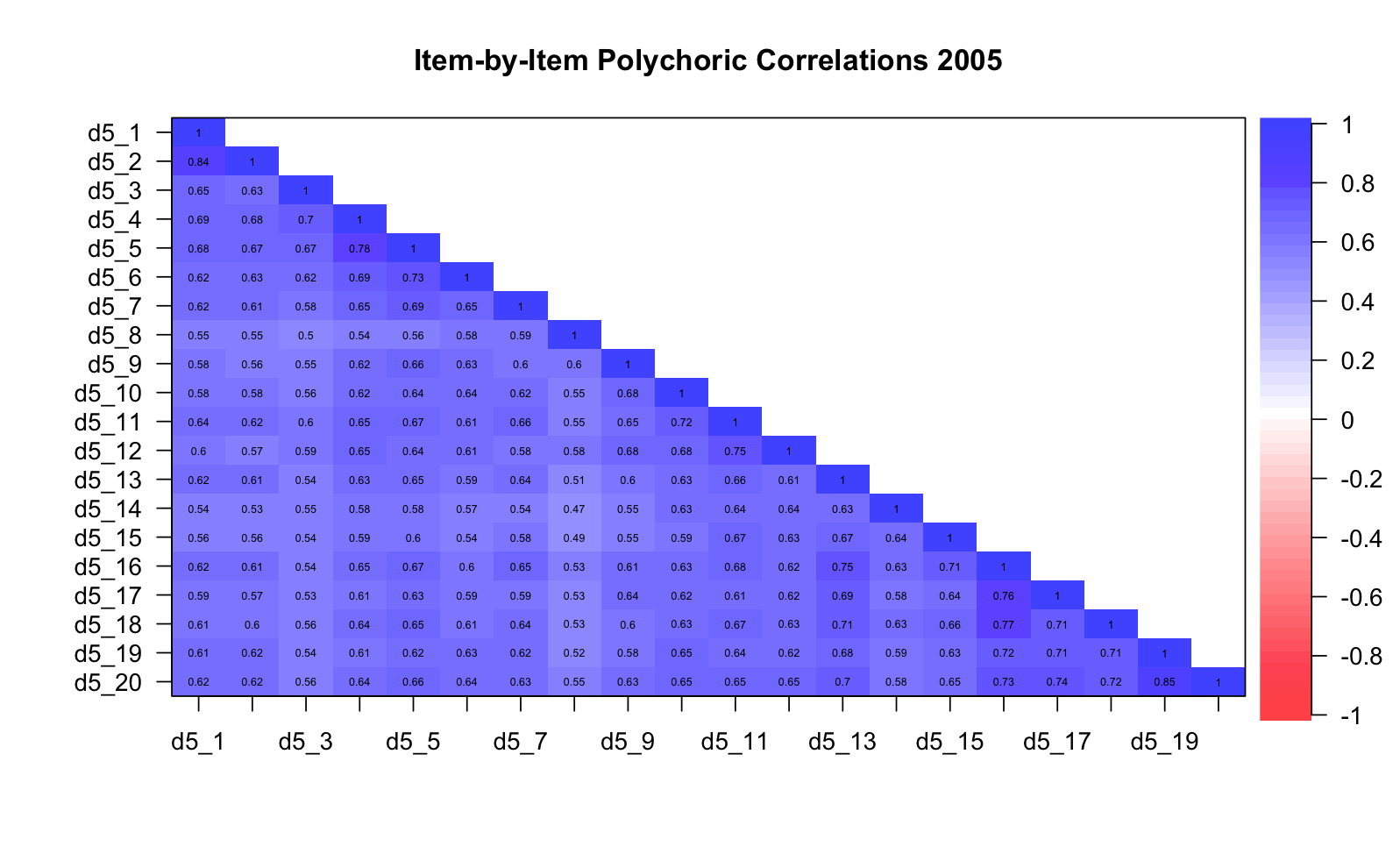


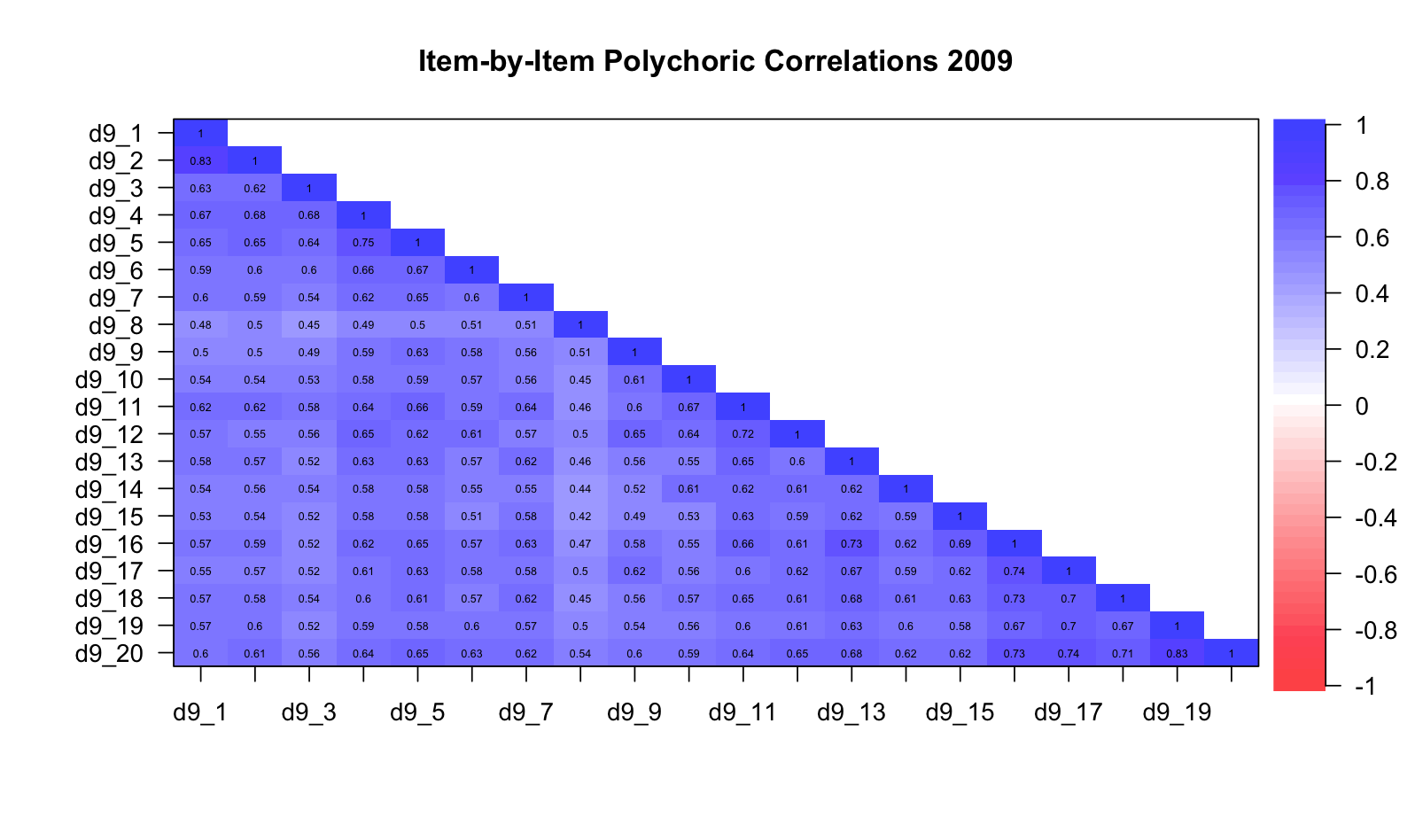


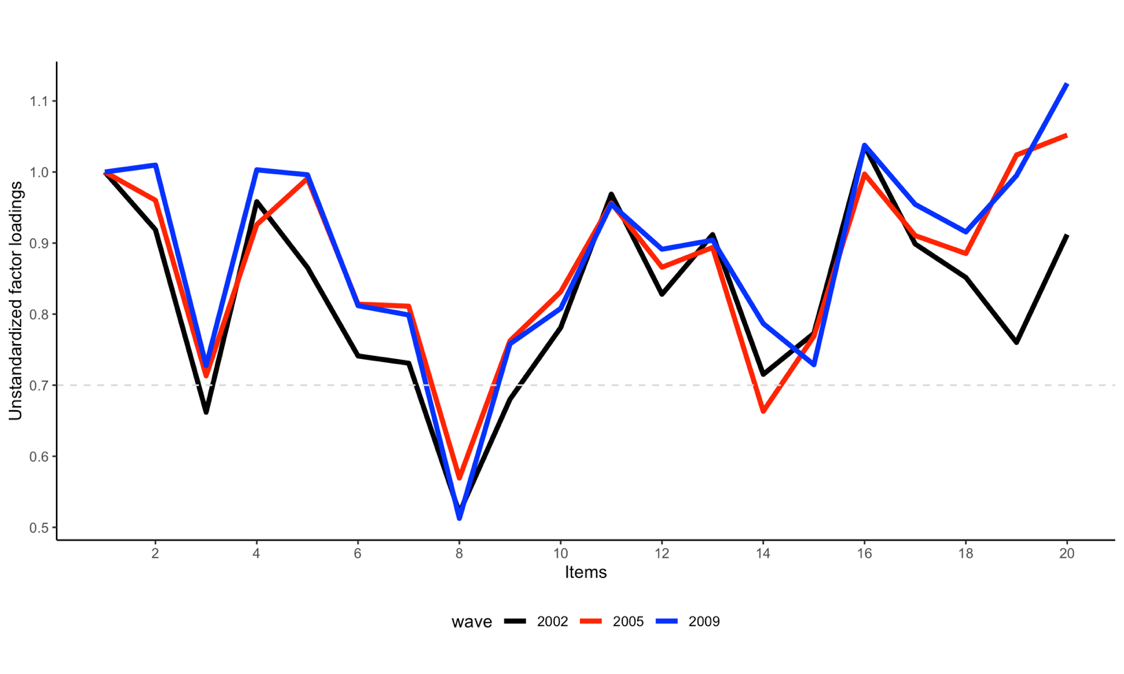


**Table S1.** Measures of fit for single CFAs for each wave using the 20-item CAL-D

| **Wave** | **Sample size** | **SB-Chisq** | **df** | **RMSEA** | **CFI** | **TLI** | **SRMR** |
| --- | --- | --- | --- | --- | --- | --- | --- |
| 2002 | 12,649 | 4772.09 | 170 | 0.046 | 0.979 | 0.977 | 0.047 |
| 2005 | 11,392 | 6904.44 | 170 | 0.059 | 0.984 | 0.982 | 0.046 |
| 2009 | 11,920 | 5180.69 | 170 | 0.050 | 0.984 | 0.983 | 0.042 |

**3. Single-wave Confirmatory Factory Analysis: Measures of fit, factor loadings, and thresholds**

We assessed model fit using absolute and comparative indices (Brown, 2015). First, we estimated a Chi-Square – the most common test to compare the theoretical with the observed models. However, given that it is highly sensitive to sample size, non-normality, and correlation size when testing measurement invariance, we estimated a Satorra-Bentler Chi-Square index which incorporates a scaling correction factor; but it is important to note that this correction effect diminishes as sample size increases (Kline, 2016). We also estimated the Root Mean Square Error of Approximation (RMSEA), an absolute fit index intended to assess the extent to which our model fitted reasonably well in the population (as opposed to the Chi-Square that tested whether the model holds exactly in the population) (Brown, 2015). Then, we calculated the Standardized Root Mean Square Residual (SRMR) to examine the average discrepancy between the observed and predicted residual correlations. In addition, we used comparative indices to examine the fit of a solution in relation to a more restricted, nested baseline model (Brown, 2015). Specifically, we estimated the Comparative Fit Index (CFI) and the Tucker-Lewis Index (TLI) to assess if the fit decreased with more restricted models. We reported all these indexes and assess their consistency using the following cutoff criteria: non-significant Chi-Square; RMSEA values below 0.06; SRMR values below 0.08; and CFI and TLI values of 0.95 or greater (Hu & Bentler, 1999). For comparison of nested models, we used the following criteria: 0.015 in ΔRMSEA; 0.01 ΔCFI and ΔTLI; and 0.03 in ΔSRMR (Putnick & Bornstein, 2016) (results of available upon request).

Single CFAs show adequate fit in every wave. Due to a large sample size, Chi-Square is always statistically significant, even when using the Satorra Bentler correction factor. Nonetheless, robust versions of the measures of fit are within the acceptable thresholds. RMSEA is 0.033 in the 2002 wave, 0.043 in the 2005 wave, and 0.034 in the 2009 wave. Moreover, CFI and TLI are all above 0.989 and SRMR is below 0.049 in every wave. Therefore, separate analyses of the CAL-DM show the scale is unidimensional, all factor loadings and thresholds are statistically significant and with similar values.

**Table S2.** Measures of fit for single CFAs for each wave using the 19-item CAL-DM

| **Wave** | **Sample size** | **SB-Chisq** | **df** | **RMSEA** | **CFI** | **TLI** | **SRMR** |
| --- | --- | --- | --- | --- | --- | --- | --- |
| 2002 | 14,264 | 5077.81 | 152 | 0.033 | 0.99 | 0.989 | 0.047 |
| 2005 | 14,180 | 9092.777 | 152 | 0.043 | 0.992 | 0.991 | 0.049 |
| 2009 | 13,974 | 6042.165 | 152 | 0.034 | 0.993 | 0.992 | 0.045 |

**Table S3.** Unstandardized and standardized factor loading coefficients for single CFAs for each wave

| Wave | **2002** | | **2005** | | | **2009** | |
| --- | --- | --- | --- | --- | --- | --- | --- |
| Item | Unstand.  coefficient | Stand  coefficient | Unstand.  coefficient | | Stand  coefficient | Unstand.  coefficient | Stand  coefficient |
| 1 | 1 | 0.788 | 1 | 0.831 | | 1 | 0.816 |
| 2 | 0.923 | 0.763 | 0.976 | 0.824 | | 1.009 | 0.818 |
| 3 | 0.67 | 0.651 | 0.723 | 0.733 | | 0.742 | 0.724 |
| 4 | 0.976 | 0.781 | 0.939 | 0.814 | | 1.014 | 0.82 |
| 5 | 0.889 | 0.751 | 0.993 | 0.829 | | 1.017 | 0.821 |
| 6 | 0.767 | 0.7 | 0.822 | 0.775 | | 0.802 | 0.75 |
| 7 | 0.742 | 0.689 | 0.811 | 0.771 | | 0.813 | 0.754 |
| 9 | 0.689 | 0.661 | 0.778 | 0.758 | | 0.76 | 0.732 |
| 10 | 0.783 | 0.708 | 0.83 | 0.778 | | 0.809 | 0.752 |
| 11 | 0.967 | 0.778 | 0.964 | 0.821 | | 0.969 | 0.807 |
| 12 | 0.837 | 0.731 | 0.867 | 0.791 | | 0.906 | 0.788 |
| 13 | 0.923 | 0.763 | 0.907 | 0.804 | | 0.929 | 0.795 |
| 14 | 0.738 | 0.686 | 0.685 | 0.715 | | 0.803 | 0.75 |
| 15 | 0.763 | 0.698 | 0.775 | 0.757 | | 0.737 | 0.721 |
| 16 | 1.039 | 0.799 | 1.021 | 0.836 | | 1.047 | 0.828 |
| 17 | 0.908 | 0.758 | 0.912 | 0.806 | | 0.948 | 0.801 |
| 18 | 0.861 | 0.74 | 0.918 | 0.808 | | 0.947 | 0.801 |
| 19 | 0.753 | 0.694 | 1.02 | 0.836 | | 1.008 | 0.818 |
| 20 | 0.92 | 0.762 | 1.058 | 0.845 | | 1.14 | 0.849 |

**Table S4.** Unstandardized threshold coefficients for single CFAs for each wave

| Item | Threshold | 2002 | 2005 | 2009 |
| --- | --- | --- | --- | --- |
| 1 | t1 | 0.285 | 0.484 | 0.495 |
| 1 | t2 | 2.532 | 3.045 | 2.739 |
| 1 | t3 | 3.54 | 3.949 | 3.659 |
| 2 | t1 | 0.481 | 0.747 | 0.69 |
| 2 | t2 | 2.529 | 3.047 | 2.822 |
| 2 | t3 | 3.622 | 4.021 | 3.763 |
| 3 | t1 | 0.304 | 0.464 | 0.38 |
| 3 | t2 | 2.092 | 2.469 | 2.27 |
| 3 | t3 | 2.962 | 3.194 | 3.101 |
| 4 | t1 | 0.526 | 0.823 | 0.942 |
| 4 | t2 | 2.795 | 3.252 | 3.076 |
| 4 | t3 | 3.97 | 4.09 | 4.006 |
| 5 | t1 | 0.895 | 1.296 | 1.212 |
| 5 | t2 | 2.879 | 3.594 | 3.21 |
| 5 | t3 | 3.837 | 4.338 | 3.984 |
| 6 | t1 | 1.021 | 1.377 | 1.241 |
| 6 | t2 | 2.613 | 3.124 | 2.838 |
| 6 | t3 | 3.458 | 3.826 | 3.567 |
| 7 | t1 | 0.818 | 1.27 | 1.137 |
| 7 | t2 | 2.464 | 2.878 | 2.741 |
| 7 | t3 | 3.158 | 3.608 | 3.41 |
| 9 | t1 | 0.71 | 0.978 | 0.835 |
| 9 | t2 | 2.277 | 2.757 | 2.439 |
| 9 | t3 | 2.91 | 3.361 | 2.987 |
| 10 | t1 | 1.128 | 1.521 | 1.321 |
| 10 | t2 | 2.666 | 3.125 | 2.856 |
| 10 | t3 | 3.53 | 3.679 | 3.375 |
| 11 | t1 | 0.304 | 0.805 | 0.802 |
| 11 | t2 | 2.524 | 3.227 | 2.913 |
| 11 | t3 | 3.675 | 4.061 | 3.659 |
| 12 | t1 | 0.286 | 0.568 | 0.56 |
| 12 | t2 | 2.382 | 2.863 | 2.644 |
| 12 | t3 | 3.459 | 3.781 | 3.501 |
| 13 | t1 | 0.919 | 1.251 | 1.24 |
| 13 | t2 | 2.843 | 3.265 | 3.108 |
| 13 | t3 | 3.611 | 3.902 | 3.804 |
| 14 | t1 | 0.537 | 0.792 | 0.801 |
| 14 | t2 | 2.244 | 2.556 | 2.523 |
| 14 | t3 | 3.104 | 3.268 | 3.252 |
| 15 | t1 | 0.524 | 0.984 | 0.871 |
| 15 | t2 | 2.333 | 2.811 | 2.626 |
| 15 | t3 | 3.343 | 3.617 | 3.272 |
| 16 | t1 | 1.077 | 1.479 | 1.444 |
| 16 | t2 | 3.155 | 3.75 | 3.39 |
| 16 | t3 | 4.13 | 4.473 | 4.083 |
| 17 | t1 | 1.204 | 1.493 | 1.406 |
| 17 | t2 | 2.857 | 3.304 | 3.023 |
| 17 | t3 | 3.714 | 4.004 | 3.699 |
| 18 | t1 | 0.797 | 1.309 | 1.138 |
| 18 | t2 | 2.715 | 3.341 | 3.104 |
| 18 | t3 | 3.686 | 4.102 | 3.801 |
| 19 | t1 | 1.746 | 2.344 | 2.193 |
| 19 | t2 | 2.934 | 3.898 | 3.495 |
| 19 | t3 | 3.406 | 4.477 | 4.148 |
| 20 | t1 | 1.608 | 2.078 | 2.048 |
| 20 | t2 | 3.07 | 3.944 | 3.72 |
| 20 | t3 | 3.662 | 4.502 | 4.437 |

**4. Measurement invariance by time**

Models comparing longitudinal invariance had adequate fit with all constraints in all wave comparisons (see Supplementary Material 4). Chi-Square was statistically significant in all models due to sample size. However, RMSEA was consistently low in all models (ranging from 0.022 to 0.024) and SRMR showed excellent values (ranging from 0.037 to 0.042). Moreover, comparative fit indices were also above the expected value; CFI in a range between 0.991 and 0.992 while TLI was between 0.991 and 0.992. Differences in the fit indices were minimal. There were no worrisome signals of misfit in the correlation residuals (all were below 0.15) or in the modification index. These results indicate the CAL-DM was invariant by time.

Table S5 Model fit indices of the invariance models for the multi-group CFA by time with the three time-waves of the MxFLS

| **Comparison of the 2002 wave with the 2005 wave (n= 16,499)** | | | | | | | | | |
| --- | --- | --- | --- | --- | --- | --- | --- | --- | --- |
| **Invariance Model** | **SB-Chisq** | **df** | **RMSEA** | **CFI** | **TLI** | **SRMR** | **dchi** | **ddf** | **P-value** |
| **Configural** | 6335.979 | 645 | 0.023 | 0.992 | 0.991 | 0.038 | - | - | - |
| **Thresholds** | 6385.992 | 664 | 0.023 | 0.992 | 0.991 | 0.038 | 105.411 | 19 | 0 |
| **Loadings** | 6438.744 | 682 | 0.023 | 0.992 | 0.991 | 0.038 | 47.345 | 18 | 0 |
| **Intercepts** | 6603.73 | 700 | 0.023 | 0.991 | 0.991 | 0.039 | 137.248 | 18 | 0 |
| **Residuals** | 6869.236 | 719 | 0.023 | 0.991 | 0.991 | 0.040 | 98.24 | 19 | 0 |
| **Comparison of the 2005 wave with the 2009 wave (n= 16,167)** | | | | | | | | | |
| **Invariance Model** | **SB-Chisq** | **df** | **RMSEA** | **CFI** | **TLI** | **SRMR** | **dchi** | **ddf** | **P-value** |
| **Configural** | 6813.734 | 645 | 0.024 | 0.992 | 0.992 | 0.039 | - | - | - |
| **Thresholds** | 6832.163 | 664 | 0.024 | 0.992 | 0.992 | 0.039 | 41.435 | 19 | 0 |
| **Loadings** | 6871.11 | 682 | 0.024 | 0.992 | 0.992 | 0.039 | 39.055 | 18 | 0 |
| **Intercepts** | 6912.32 | 700 | 0.023 | 0.992 | 0.992 | 0.039 | 36.675 | 18 | 0.01 |
| **Residuals** | 7112.302 | 719 | 0.023 | 0.992 | 0.992 | 0.040 | 76.003 | 19 | 0 |
| **Comparison of the 2002 wave with the 2009 wave (n= 16,475)** | | | | | | | | | |
| **Invariance Model** | **SB-Chisq** | **df** | **RMSEA** | **CFI** | **TLI** | **SRMR** | **dchi** | **ddf** | **P-value** |
| **Configural** | 5850.211 | 645 | 0.022 | 0.992 | 0.992 | 0.037 | - | - | - |
| **Thresholds** | 5881.131 | 664 | 0.022 | 0.992 | 0.992 | 0.037 | 64.144 | 19 | 0 |
| **Loadings** | 5898.7 | 682 | 0.022 | 0.992 | 0.992 | 0.037 | 16.164 | 18 | 0.58 |
| **Intercepts** | 6069.45 | 700 | 0.022 | 0.992 | 0.992 | 0.037 | 138.454 | 18 | 0 |
| **Residuals** | 6231.06 | 719 | 0.022 | 0.992 | 0.992 | 0.038 | 59.019 | 19 | 0 |

**Note:** We report robust fit indices. *SB-Chisq*= Satorra-Bentler Chi-square; *df*= degrees of freedom; *RMSEA*= Root Mean Square Error of Approximation; *CFI*= Comparative Fit Index; *TLI*= Tucker-Lewis Index; *SRMR*= Standardized Root Mean Square Residual; *dchi*= Chi-square difference; *ddf*= difference in degrees of freedom; *P-value*= P-value of the Chi-square difference.

Longitudinal invariance holds when measured simultaneously in the three waves (see Table S5). These results indicate that longitudinal comparisons with the 19-item scale with observed composite scores are warranted.

Table S6. Model fit indices of the time invariance models comparing the three waves of the MxFLS (n= 16,868)

| **Invariance Model** | **SB-Chisq** | **df** | **RMSEA** | **CFI** | **TLI** | **SRMR** | **dchi** | **ddf** | **P-value** |
| --- | --- | --- | --- | --- | --- | --- | --- | --- | --- |
| **Configural** | 10131.86 | 1479 | 0.019 | 0.992 | 0.992 | 0.035 | - | - | - |
| **Thresholds** | 10202.55 | 1517 | 0.018 | 0.992 | 0.992 | 0.035 | 151.885 | 38 | 0 |
| **Loadings** | 10272.35 | 1553 | 0.018 | 0.992 | 0.992 | 0.035 | 63.918 | 36 | 0 |
| **Intercepts** | 10534.62 | 1589 | 0.018 | 0.992 | 0.992 | 0.035 | 208.892 | 36 | 0 |
| **Residuals** | 10992.32 | 1627 | 0.018 | 0.992 | 0.992 | 0.036 | 143.166 | 38 | 0 |

**Note:** We report robust fit indices. *SB-Chisq*= Satorra-Bentler Chi-square; *df*= degrees of freedom; *RMSEA*= Root Mean Square Error of Approximation; *CFI*= Comparative Fit Index; *TLI*= Tucker-Lewis Index; *SRMR*= Standardized Root Mean Square Residual; *dchi*= Chi-square difference; *ddf*= difference in degrees of freedom; *P-value*= P-value of the Chi-square difference.

**5. Measurement invariance by sex and time by pair of waves**

These results show adequate fit in all pairs of models demonstrating sex and time invariance simultaneously. Consistent with previous results, the 19-item CAL-DM scale had adequate fit. Chi-Square remained significant in all models. As expected, RMSEA was low, ranging from 0.021 to 0.025, and SRMR from 0.042 to 0.047. Comparative indices had high values; CFI ranged between 0.989 and 0.992 and TLI between 0.989 and 0.992. Differences in model fit were negligible and there were no concerning points of local misfit. Results for each pair of waves are available in Table S6.

**Table S7.** Model fit indices of the invariance models by time and sex

| **Comparison of the 2002 wave with the 2005 wave with sex** (N= 16,499) | | | | | | | | | |
| --- | --- | --- | --- | --- | --- | --- | --- | --- | --- |
| **Invariance Model** | **SB-Chisq** | **df** | **RMSEA** | **CFI** | **TLI** | **SRMR** | **dchi** | **ddf** | **P-value** |
| **Configural** | 7173.445 | 1290 | 0.024 | 0.991 | 0.990 | 0.044 | - | - | - |
| **Thresholds** | 7253.256 | 1347 | 0.023 | 0.991 | 0.990 | 0.044 | 173.647 | 57 | 0 |
| **Loadings** | 7381.426 | 1401 | 0.023 | 0.991 | 0.991 | 0.044 | 112.735 | 54 | 0 |
| **Intercepts** | 7919.081 | 1455 | 0.023 | 0.990 | 0.990 | 0.044 | 413.314 | 54 | 0 |
| **Residuals** | 8753.601 | 1512 | 0.024 | 0.989 | 0.989 | 0.047 | 314.381 | 57 | 0 |
| **Comparison of the 2005 wave with the 2009 wave with sex** (N= 16,167) | | | | | | | | | |
| **Invariance Model** | **SB-Chisq** | **df** | **RMSEA** | **CFI** | **TLI** | **SRMR** | **dchi** | **ddf** | **P-value** |
| **Configural** | 7617.905 | 1290 | 0.025 | 0.992 | 0.991 | 0.043 | - | - | - |
| **Thresholds** | 7671.232 | 1347 | 0.024 | 0.992 | 0.992 | 0.043 | 126.583 | 57 | 0 |
| **Loadings** | 7787.724 | 1401 | 0.024 | 0.992 | 0.992 | 0.044 | 119.493 | 54 | 0 |
| **Intercepts** | 8194.519 | 1455 | 0.024 | 0.991 | 0.992 | 0.044 | 346.288 | 54 | 0 |
| **Residuals** | 9004.18 | 1512 | 0.025 | 0.990 | 0.991 | 0.045 | 312.906 | 57 | 0 |
| **Comparison of the 2002 wave with the 2009 wave** (N= 16,475) | | | | | | | | | |
| **Invariance Model** | **SB-Chisq** | **df** | **RMSEA** | **CFI** | **TLI** | **SRMR** | **dchi** | **ddf** | **P-value** |
| **Configural** | 6508.118 | 1290 | 0.022 | 0.992 | 0.991 | 0.042 | - | - | - |
| **Thresholds** | 6554.981 | 1347 | 0.022 | 0.992 | 0.991 | 0.042 | 97.875 | 57 | 0.001 |
| **Loadings** | 6640.465 | 1401 | 0.021 | 0.992 | 0.992 | 0.042 | 74.904 | 54 | 0.031 |
| **Intercepts** | 7284.087 | 1455 | 0.022 | 0.991 | 0.991 | 0.043 | 480.214 | 54 | 0 |
| **Residuals** | 7860.439 | 1512 | 0.023 | 0.990 | 0.991 | 0.045 | 219.878 | 57 | 0 |

**Note:** We report robust fit indices. *SB-Chisq*= Satorra-Bentler Chi-square; *df*= degrees of freedom; *RMSEA*= Root Mean Square Error of Approximation; *CFI*= Comparative Fit Index; *TLI*= Tucker-Lewis Index; *SRMR*= Standardized Root Mean Square Residual; *dchi*= Chi-square difference; *ddf*= difference in degrees of freedom; *P-value*= P-value of the Chi-square difference.

**Factor Loading and threshold parameters**

Table S8. Unstandardized factor loading coefficients for the fully constrained invariance model in a multi-group CFA by sex and time

| Item | Unstandardized  coefficient | Std. Error | Confidence interval | |
| --- | --- | --- | --- | --- |
| 1 | 1.166 | 0.032 | 1.103 | 1.229 |
| 2 | 1.162 | 0.032 | 1.1 | 1.225 |
| 3 | 0.833 | 0.023 | 0.787 | 0.878 |
| 4 | 1.149 | 0.031 | 1.087 | 1.21 |
| 5 | 1.144 | 0.033 | 1.079 | 1.208 |
| 6 | 0.933 | 0.029 | 0.877 | 0.99 |
| 7 | 0.940 | 0.027 | 0.887 | 0.994 |
| 9 | 0.869 | 0.024 | 0.823 | 0.915 |
| 10 | 0.960 | 0.029 | 0.904 | 1.016 |
| 11 | 1.14 | 0.031 | 1.08 | 1.2 |
| 12 | 1.034 | 0.028 | 0.98 | 1.089 |
| 13 | 1.08 | 0.03 | 1.021 | 1.139 |
| 14 | 0.874 | 0.025 | 0.824 | 0.924 |
| 15 | 0.892 | 0.026 | 0.84 | 0.943 |
| 16 | 1.217 | 0.035 | 1.148 | 1.286 |
| 17 | 1.094 | 0.032 | 1.032 | 1.157 |
| 18 | 1.056 | 0.03 | 0.997 | 1.116 |
| 19 | 1.115 | 0.036 | 1.045 | 1.186 |
| 20 | 1.228 | 0.037 | 1.155 | 1.301 |

**Note**: Item 8 was excluded from all analyses. Std. error= Standard error

Table S9. Unstandardized threshold coefficients for the fully constrained invariance model in a multi-group CFA by sex and time

| Item | Threshold | Unstandardized  coefficient | Std. Error | Confidence interval | |
| --- | --- | --- | --- | --- | --- |
| 1 | t1 | 0.583 | 0.033 | 0.518 | 0.648 |
| 1 | t2 | 2.919 | 0.044 | 2.833 | 3.004 |
| 1 | t3 | 3.871 | 0.052 | 3.769 | 3.973 |
| 2 | t1 | 0.801 | 0.034 | 0.734 | 0.868 |
| 2 | t2 | 2.964 | 0.045 | 2.876 | 3.051 |
| 2 | t3 | 3.990 | 0.053 | 3.886 | 4.093 |
| 3 | t1 | 0.488 | 0.025 | 0.439 | 0.538 |
| 3 | t2 | 2.364 | 0.034 | 2.298 | 2.430 |
| 3 | t3 | 3.187 | 0.043 | 3.103 | 3.270 |
| 4 | t1 | 0.905 | 0.034 | 0.838 | 0.971 |
| 4 | t2 | 3.162 | 0.044 | 3.075 | 3.248 |
| 4 | t3 | 4.137 | 0.054 | 4.031 | 4.243 |
| 5 | t1 | 1.280 | 0.037 | 1.209 | 1.352 |
| 5 | t2 | 3.343 | 0.048 | 3.249 | 3.437 |
| 5 | t3 | 4.186 | 0.058 | 4.074 | 4.299 |
| 6 | t1 | 1.333 | 0.032 | 1.271 | 1.395 |
| 6 | t2 | 2.975 | 0.044 | 2.889 | 3.061 |
| 6 | t3 | 3.718 | 0.057 | 3.606 | 3.830 |
| 7 | t1 | 1.180 | 0.031 | 1.120 | 1.240 |
| 7 | t2 | 2.826 | 0.039 | 2.749 | 2.904 |
| 7 | t3 | 3.489 | 0.049 | 3.394 | 3.584 |
| 9 | t1 | 0.941 | 0.027 | 0.888 | 0.993 |
| 9 | t2 | 2.552 | 0.034 | 2.486 | 2.618 |
| 9 | t3 | 3.153 | 0.040 | 3.074 | 3.232 |
| 10 | t1 | 1.465 | 0.034 | 1.399 | 1.532 |
| 10 | t2 | 3.022 | 0.045 | 2.934 | 3.111 |
| 10 | t3 | 3.659 | 0.054 | 3.554 | 3.764 |
| 11 | t1 | 0.774 | 0.033 | 0.709 | 0.840 |
| 11 | t2 | 3.031 | 0.043 | 2.945 | 3.116 |
| 11 | t3 | 3.977 | 0.052 | 3.875 | 4.078 |
| 12 | t1 | 0.595 | 0.030 | 0.537 | 0.654 |
| 12 | t2 | 2.771 | 0.038 | 2.696 | 2.845 |
| 12 | t3 | 3.737 | 0.049 | 3.640 | 3.833 |
| 13 | t1 | 1.268 | 0.034 | 1.202 | 1.334 |
| 13 | t2 | 3.209 | 0.044 | 3.124 | 3.295 |
| 13 | t3 | 3.909 | 0.053 | 3.805 | 4.013 |
| 14 | t1 | 0.813 | 0.027 | 0.759 | 0.866 |
| 14 | t2 | 2.574 | 0.036 | 2.504 | 2.643 |
| 14 | t3 | 3.356 | 0.044 | 3.269 | 3.443 |
| 15 | t1 | 0.890 | 0.028 | 0.836 | 0.945 |
| 15 | t2 | 2.671 | 0.037 | 2.599 | 2.744 |
| 15 | t3 | 3.511 | 0.047 | 3.419 | 3.603 |
| 16 | t1 | 1.488 | 0.039 | 1.411 | 1.564 |
| 16 | t2 | 3.548 | 0.052 | 3.446 | 3.650 |
| 16 | t3 | 4.368 | 0.064 | 4.243 | 4.492 |
| 17 | t1 | 1.503 | 0.037 | 1.431 | 1.576 |
| 17 | t2 | 3.195 | 0.046 | 3.104 | 3.286 |
| 17 | t3 | 3.888 | 0.055 | 3.781 | 3.996 |
| 18 | t1 | 1.184 | 0.034 | 1.117 | 1.251 |
| 18 | t2 | 3.139 | 0.045 | 3.051 | 3.227 |
| 18 | t3 | 3.928 | 0.055 | 3.821 | 4.035 |
| 19 | t1 | 2.264 | 0.049 | 2.168 | 2.361 |
| 19 | t2 | 3.603 | 0.061 | 3.484 | 3.722 |
| 19 | t3 | 4.192 | 0.069 | 4.058 | 4.327 |
| 20 | t1 | 2.051 | 0.047 | 1.958 | 2.144 |
| 20 | t2 | 3.701 | 0.061 | 3.582 | 3.820 |
| 20 | t3 | 4.334 | 0.068 | 4.200 | 4.467 |

**Note**: Item 8 was excluded from all analyses. Std. error= Standard error
